# ICOS-Targeted Peptide Imaging Informs Therapeutic Response of STING Agonist in Lung Adenocarcinoma

**DOI:** 10.2967/jnumed.125.271193

**Published:** 2026-07

**Authors:** Shao Duan, Boyu Tan, Yuqiang Mao, Yue Zhang, Hongyue Lou, Xiaonan Wei, Chunrong Qu, Chao Li, Chengwei Jing, Yifei Xia, Zengping Duan, Zhen Cheng, Chuanliu Wu, Zunyu Xiao

**Affiliations:** 1 Department of Nuclear Medicine, First Affiliated Hospital of Ningbo University, Ningbo, China;; 2 State Key Laboratory of Drug Research, Molecular Imaging Center, Shanghai Institute of Materia Medica, Chinese Academy of Sciences, Shanghai, China;; 3 Department of Orthopedics, Second Affiliated Hospital of Harbin Medical University, Harbin, China;; 4 School of Life Science and Technology, ShanghaiTech University, Shanghai, China;; 5 Department of Thoracic Surgery, Shengjing Hospital of China Medical University, Shenyang, China; and; 6 MOE Key Laboratory of Spectrochemical Analysis and Instrumentation, Department of Chemistry, College of Chemistry and Chemical Engineering, Xiamen University, Xiamen, China

**Keywords:** ICOS peptide, T cells, immunotherapy, PET/CT, STING agonist

## Abstract

The innovation of cancer immunotherapy is improving clinical theranostics for lung cancer. Given the variation of therapeutic effects among patients, there is an urgent need to develop novel tools for precisely evaluating immune responses. **Methods:** Herein, we first described the potential of the inducible costimulator (ICOS) as a promising imaging biomarker for assessing cancer immunotherapy through multiple omics datasets and subsequently developed ^68^Ga-DOTA-ICOSpep, a novel peptide-based PET tracer targeting human ICOS. We characterized ICOS expression patterns via flow cytometry and immunofluorescence and performed ^68^Ga-DOTA-ICOSpep PET imaging and cytokine testing on humanized A549 cell line–derived mouse models receiving cyclic guanosine monophosphate–adenosine monophosphate and diABZI stimulator of interferon genes (STING) agonist treatment. **Results:** ^68^Ga-DOTA-ICOSpep could capture human ICOS-positive activated T cells with high specificity in vivo, which was validated via linear regression analysis between tumor PET region-of-interest quantifications against ICOS immunohistochemistry staining as well as a CD3 depletion study. **Conclusion:** ICOS PET imaging enabled precise evaluation of therapeutic responses and increased proinflammatory cytokine release induced by the STING agonist. Our data demonstrated that ICOS is a robust biomarker for assessing immune responses, and ^68^Ga-DOTA-ICOSpep PET imaging is a reliable tool for predicting and monitoring therapeutic effects of cancer immunotherapy in lung adenocarcinoma.

## Visual Abstract

**Figure:**
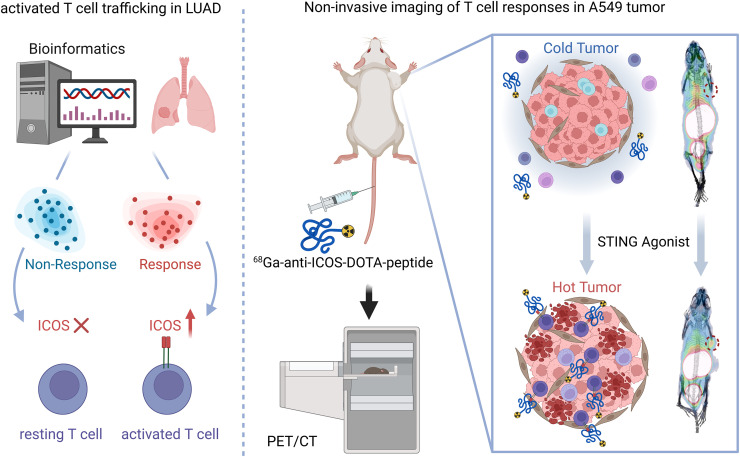

Lung adenocarcinoma (LUAD) is one of the most aggressive and fatal tumor types, with an overall survival of less than 5 y (1,2). Antitumor immunity plays a crucial role in LUAD therapy, and emerging technologies are advancing our understanding of immuno-oncology (3). Emerging immune checkpoints and costimulatory molecules (e.g., inducible T cell costimulator [ICOS] ligand, OX40, 4-1BB, CD27, CD28) are currently under investigation as potential therapeutic targets. However, patient responses remain limited because of treatment resistance. Treatment resistance in LUAD has been attributed to several factors, including systemic immunosuppression, an immunosuppressive tumor microenvironment (TME), and tumor-induced sequestration of T cells in the bone marrow (4). T cell types within the TME are the most prevalent stromal cell types in malignancies and surrounding lung tissues (5). Tumor-infiltrating T cells primarily exhibit fatigued and regulatory T cell characteristics. Suppressed T cells cannot effectively destroy tumor cells or activate adaptive immunity (6). As a result, strategies aimed at enhancing intratumoral T cell density are being actively explored to improve therapeutic efficacy. The stimulator of interferon genes (STING) signaling pathway plays an important role in the immunotherapy of LUAD (7). Systemic administration of STING agonists in mice eliminates latent metastases and prevents spontaneous tumor recurrence in a manner dependent on both T cells and natural killer cells (8). An ideal monitoring tool for patients receiving immunotherapy would assess the immune response itself, with activated T cells being of particular importance.

Circulating biomarkers, such as blood cells and exosomes, provide valuable information about immune status (9). However, blood- and serum-based indicators offer limited insights into the spatial distribution and interactions of key immune effector cells. Tissue analysis typically relies on clinical biopsies or necropsies in preclinical models. Although biopsies can assess the number and phenotype of ICOS-positive T cells, they are limited in their ability to capture spatial heterogeneity or track temporal changes in cell localization. In contrast, noninvasive molecular imaging techniques, such as PET, enable real-time visualization of immune system components in living organisms during disease progression and in response to immunotherapy (10). Immuno-PET, which combines the specificity of antibodies with the imaging benefits of PET, is thus an appealing choice (11). Because of the limited specificity of PET tracers for activated T cells, recent efforts have focused on imaging probes targeting T cell costimulatory receptors. ICOS, a CD28 superfamily member predominantly expressed on activated T cells, serves as a promising biomarker. ICOS-targeted imaging can effectively predict T cell–mediated immune responses in Lewis lung cancer models and in CD19 chimeric antigen receptor-T therapy (12,13). Rapid in vivo clearance of peptides may offer better choices for optimization of T cell imaging (14). In the current study, we developed a novel PET tracer based on the ICOS-targeting peptide (15). The ICOS peptide was labeled with ^68^Ga, and the binding affinity and specificity were validated via cell uptake and surface plasmon resonance studies. In vivo PET imaging studies demonstrated the capability of ^68^Ga-DOTA-ICOSpep to noninvasively capture ICOS-positive activated T cells in a humanized A549 mouse model receiving STING agonist therapy. Using this approach, we could precisely predict and monitor T cell–mediated therapeutic responses in LUAD models, which warrants further evaluation in the clinic.

## MATERIALS AND METHODS

All study data are available in the main text or the supplemental materials (supplemental materials are available at http://jnm.snmjournals.org).

## RESULTS

### ICOS Is Highly Related to Immunotherapy Response and Prolonged Survival in Patients with LUAD

**FIGURE 1.**
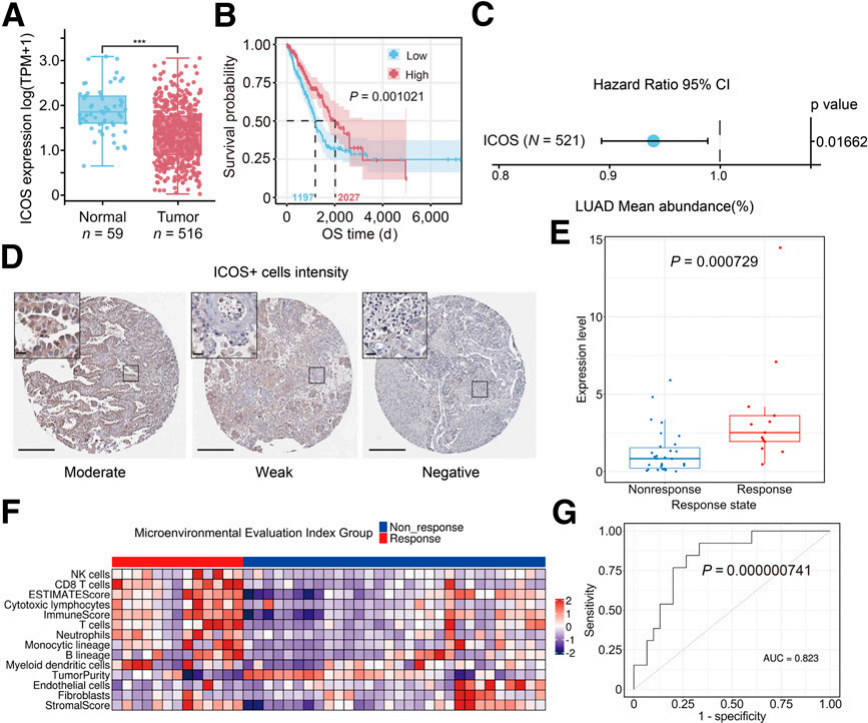
ICOS is indicator highly related to immunotherapy response and prolonged survival in patients with LUAD. (A) Box plots illustrate differential messenger RNA expression levels of ICOS between tumor and normal tissues in LUAD. (B) Kaplan–Meier survival analysis comparing prognosis between high and low ICOS expression groups. (C) Univariate Cox regression analysis identifying ICOS as prognostic factor in LUAD. (D) Immunohistochemical validation of ICOS protein expression in LUAD tissue (scale bar, 200 μm; 50 μm). (E) Differential expression of ICOS between responder and nonresponder groups in patients with LUAD receiving immunotherapy (Nivolumab and programmed death protein-1/programmed death ligand-1 blockade). (F) TME analysis depicted as heatmap illustrating immune cell infiltration profiles. (G) Receiver-operating-characteristic curve evaluating diagnostic performance of ICOS, with corresponding area under curve (AUC) values. All values represent mean ± SEM unless otherwise specified. One-way ANOVA and unpaired 2-tailed Student *t* test were used for comparation of ROI values. ***P < 0.001. NK = natural killer; OS = overall survival.

To evaluate ICOS expression in patients with LUAD, omics datasets from TCGA (The Cancer Genome Atlas Program) were first used for analysis. Significant downregulation of ICOS messenger RNA expression was observed in LUAD tissues, compared with their corresponding normal tissues (*P* < 0.001) (Fig. 1A), which may be attributed to the immunosuppressive TME and impaired infiltration of activated T cells. Additional independent transcriptomic datasets from TCGA demonstrated robust association between ICOS expression and patient survival. Kaplan–Meier analysis revealed that LUAD patients with high ICOS expression exhibited significantly longer survival (*P* = 0.001021), compared with those who had low ICOS expression (Fig. 1B). Further, univariate Cox proportional hazards regression demonstrated that ICOS expression was significantly associated with overall survival in patients with LUAD (coefficient, −0.06227; hazard ratio, 0.94), identifying ICOS as a protective factor (*P* = 0.01662) (Fig. 1C). In line with TCGA datasets, immunohistochemical staining from the Human Protein Atlas datasets showed sparse ICOS-positive cell intensity from negative to moderate ICOS expression in LUAD tissues, indicating that ICOS is an ideal imaging biomarker with low background in LUAD (Fig. 1D). To verify whether ICOS could be an indicator for assessing therapeutic response of LUAD immunotherapy, the Cancer Treatment Response gene signature database was also used. ICOS gene expression is significantly higher in the responder group than in the nonresponder group (*P* = 0.000729) (Fig. 1E). The increased ICOS expression may be related to activated T cell infiltration in the TME (Fig. 1F). To test the ability of ICOS to predict immunotherapy responses, receiver-operating-characteristic curve analysis was performed, and ICOS had excellent predictive potential for selecting immunotherapy responders (area under the curve, 0.82; *P* = 0.000000741) (Fig. 1G). The above omics datasets demonstrated that ICOS is a protective factor and an indicator for assessing therapeutic response of immunotherapy in LUAD.

### Evaluation of ICOS Expression in Humanized A549 Cell Line–Derived (CDX) Models Receiving STING Agonist Therapy

**FIGURE 2.**
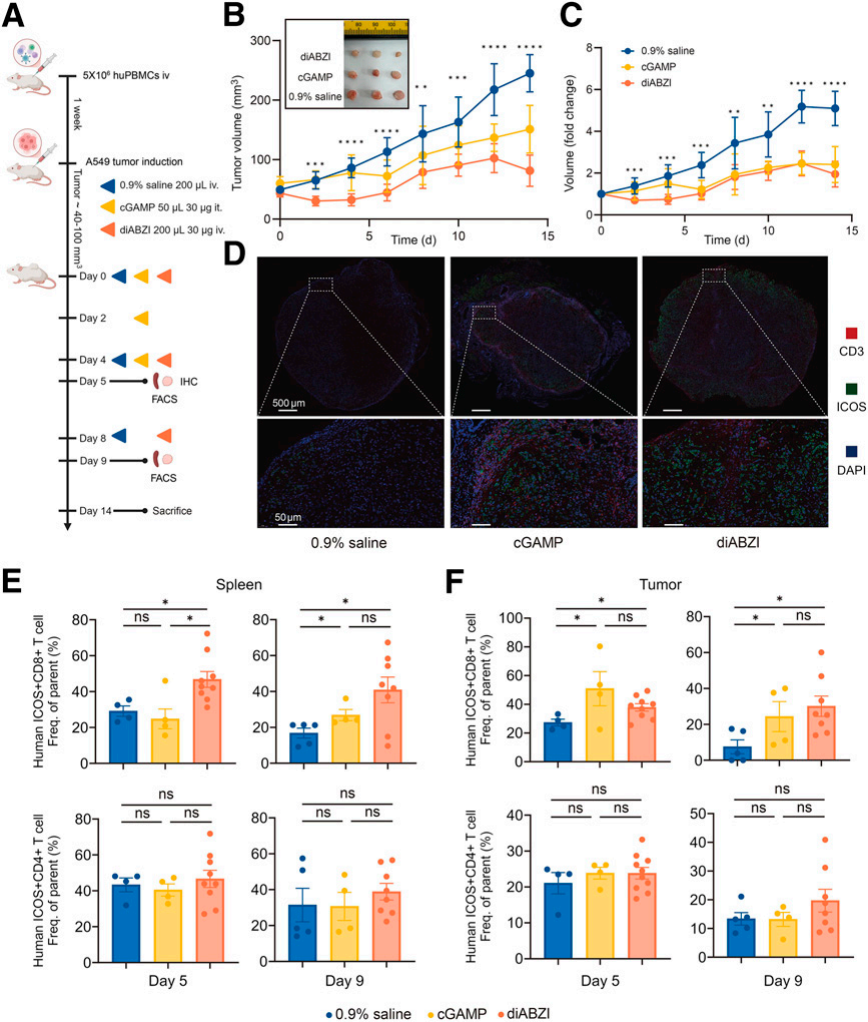
ICOS serves as specific marker of activated T cells in LUAD. (A) Scheme of ICOS targeting in humanized A549 tumor–bearing mice. Blue, yellow, and orange arrows represent groups and administration time points on timeline. Day 5: immunohistochemistry. Days 5 and 14: flow cytometry analysis; sacrifice of mice in accordance with animal ethics committee. (B) Tumor volume monitoring in mice treated with 0.9% saline, intratumoral cGAMP, or intravenous diABZI (day 14 tumor photo). (C) Tumor fold change (days 2, 4, 6, 8, 10, 12, and 14 tumor volume/day 0 tumor volume). (D) Day 5: immunofluorescence staining of A549 tumors after treatment, CD3 (red, pan-T cell marker), ICOS (green, activated T cell marker), and 4′,6-diamidino-2-phenylindole (DAPI) (blue, nuclear stain). (E and F) Flow cytometry analysis of ICOS expression on T cells isolated from tumors and spleens at early and late stages. All values represent mean ± SEM unless otherwise specified. One-way ANOVA and unpaired 2-tailed Student *t* test were used for comparation of ROI values. *P < 0.05, **P < 0.01, ***P < 0.001, and ****P < 0.0001. FACS = fluorescence-activated cell sorting; huPBMCs = humanized peripheral blood mononuclear cells; IHC = immunohistochemistry; iv = intravenous; ns = not significant.

STING is an endoplasmic protein that mainly induces the production of type I interferon, making it an attractive target for cancer immunotherapy, and in fact, there have been numerous natural and synthetic STING agonists assessed in various cancers. In the current study, we used 2 types of STING agonists as immunotherapy adjuvants, cyclic guanosine monophosphate–adenosine monophosphate (cGAMP) (intratumoral injection) or diABZI (systemic administration). Humanized peripheral blood mononuclear cell (PBMC) A549-derived CDX mouse models were selected for assessment. The whole animal study scheme is presented in Figure 2A. The A549 mice received 3 doses of either 0.9% saline, cGAMP, or diABZI, and tumor volumes were recorded every other day. Both cGAMP and diABZI groups exhibited slower tumor growth and lower fold change in tumor volume compared with the saline group (Figs. 2B and 2C; Supplemental Fig. 1A), whereas there was no difference between the 2 STING agonist groups. At day 5, tumors from different groups were collected, and immunofluorescence staining was performed. ICOS was highly expressed in both cGAMP and diABZI groups, whereas the 0.9% saline group exhibited an immune-desert phenotype (Fig. 2D; Supplemental Figs. 1B and 1C). In the cGAMP and diABZi groups, the expression of CD3 and ICOS was higher and exhibited colocalization, indicating significant infiltration of activated T cells. Good colocalization was also observed between CD3 and ICOS, indicating that ICOS was mainly expressed on T cells, and this was consistent with previous findings (16). To further confirm the feasibility of ICOS as an imaging biomarker for predicting the response of immunotherapy, a flow cytometry analysis study was conducted, and the gating strategy is listed in Supplemental Figure 2A. Two weeks after PBMC implantation, the T cells had completely integrated (Supplemental Fig. 2B). From early-stage day 5, a higher frequency of ICOS-positive CD8^+^ T cells in the spleen was detected in the diABZI cohort compared with that in both cGAMP and 0.9% saline groups (*P* < 0.05), and this may be attributed to the systemic immune response induced by systemic administration of diABZI. From late-stage day 9, ICOS-positive CD8^+^ T cells in the spleen from both cGAMP and diABZI cohorts were higher than that in the 0.9% saline group (*P* < 0.05) (Fig. 2E). In tumors, a higher frequency of ICOS-positive CD8^+^ T cells could be observed from both cGAMP- and diABZI-treated mice compared with 0.9% saline group (*P* < 0.05) (Fig. 2F) at both early and late stage; however, no difference of ICOS-positive CD4^+^ T cells could be detected, indicating that the STING agonist activates an adaptive immune response that mainly relies on inducing cytotoxic T cells (17,18). Immunofluorescence staining and fluorescence-activated cell sorting data demonstrated that with STING agonist therapy, ICOS was upregulated in both tumors and the spleen, indicating ICOS as a sensitive biomarker for assessing the response of cancer immunotherapy from both local (tumor) and systemic (spleen) locations.

### Synthesis and In Vitro Evaluation of a ^68^Ga-Labeled ICOS-Targeted PET Probe

**FIGURE 3.**
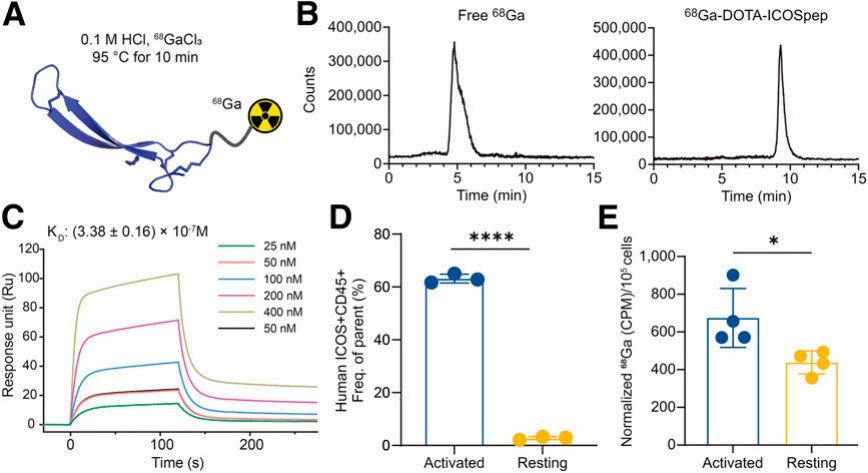
Synthesis, characterization, and in vitro cell uptake study of ^68^Ga-DOTA-ICOSpep. (A) Chemical structure and ^68^Ga labeling of DOTA-ICOS peptide. (B) Radio–high-performance liquid chromatography analysis of ^68^Ga-DOTA-ICOSpep, with separation from free ^68^Ga. (C) Surface plasmon resonance assay demonstrating binding affinity of DOTA-ICOS peptide to recombinant human ICOS protein. (D) Flow cytometry analysis of ICOS expression on activated (phorbol 12-myristate 13-acetate and ionomycin incubation for 24 h) versus resting T cells. (E) Cellular uptake assay comparing accumulation of ^68^Ga-DOTA-ICOSpep in activated and resting T cells. All values represent mean ± SEM unless otherwise specified. One-way ANOVA and unpaired 2-tailed Student *t* test were used for comparation of ROI values. *P < 0.05 and ****P < 0.0001. CPM = counts per minute; K_D_ = dissociation constant.

The ICOS-targeting peptide was synthesized according to the standard protocol of solid-phase peptide synthesis, with the peptide sequence listed in Supplemental Figure 3A. DOTA was conjugated to the lysine side chains of the peptide. For chemical characterization, high-performance liquid chromatography and mass spectrometry were performed. The purity of the ICOS peptide was greater than 99%, and the molecular weight was 4426.875. Ten micrograms of peptide (2.26 nmol) were labeled with approximately 37 MBq (1 mCi) of ^68^Ga. This corresponds to a molar activity of approximately 16.4 MBq/nmol at the end of the synthesis (Supplemental Figs. 3B and 3C). For radiochemistry, DOTA-ICOSpep was radiolabeled with ^68^GaCl_3_ at 95 °C (Fig. 3A), with a yield of greater than 70% based on 4 validation runs. The radiochemical purity was greater than 99% via instant thin-layer chromatography validation (Fig. 3B). Surface plasmon resonance assay demonstrated strong binding affinity of the ICOS peptide (unconjugated) to the recombinant human ICOS protein, with a dissociation constant of (3.38 ± 0.16) × 10^−7^ M (Fig. 3C; Supplemental Fig. 3D). ^68^Ga-DOTA-ICOSpep stability was also tested in phosphate-buffered saline, fetal bovine serum, and human serum albumin (Supplemental Fig. 3E). To evaluate the specificity of ^68^Ga-DOTA-ICOSpep on activated human T cells, human PBMCs (1 × 10^6^ cells) were stimulated with a phorbol 12-myristate 13-acetate/ionomycin cocktail, and ICOS expression was validated via flow cytometry. ICOS was strongly upregulated on activated T cells compared with resting human T cells (*P* < 0.0.0001) (Fig. 3D). For the cell uptake study, ^68^Ga-DOTA-ICOSpep (radioactive activity, 185 kBq [5 μCi]; mass, 0.1 μg per well) was incubated with either activated or resting human T cells (1 × 10^6^ cells per well; *n* = 6) for 60 min, and higher radioactivity could be detected in activated T cells (*P* < 0.05) (Fig. 3E), demonstrating the high specificity of ^68^Ga-DOTA-ICOSpep to activated human T cells.

### ^68^Ga-DOTA-ICOSpep PET Imaging Identifies T Cell–Mediated Immune Responses to STING Agonist

**FIGURE 4.**
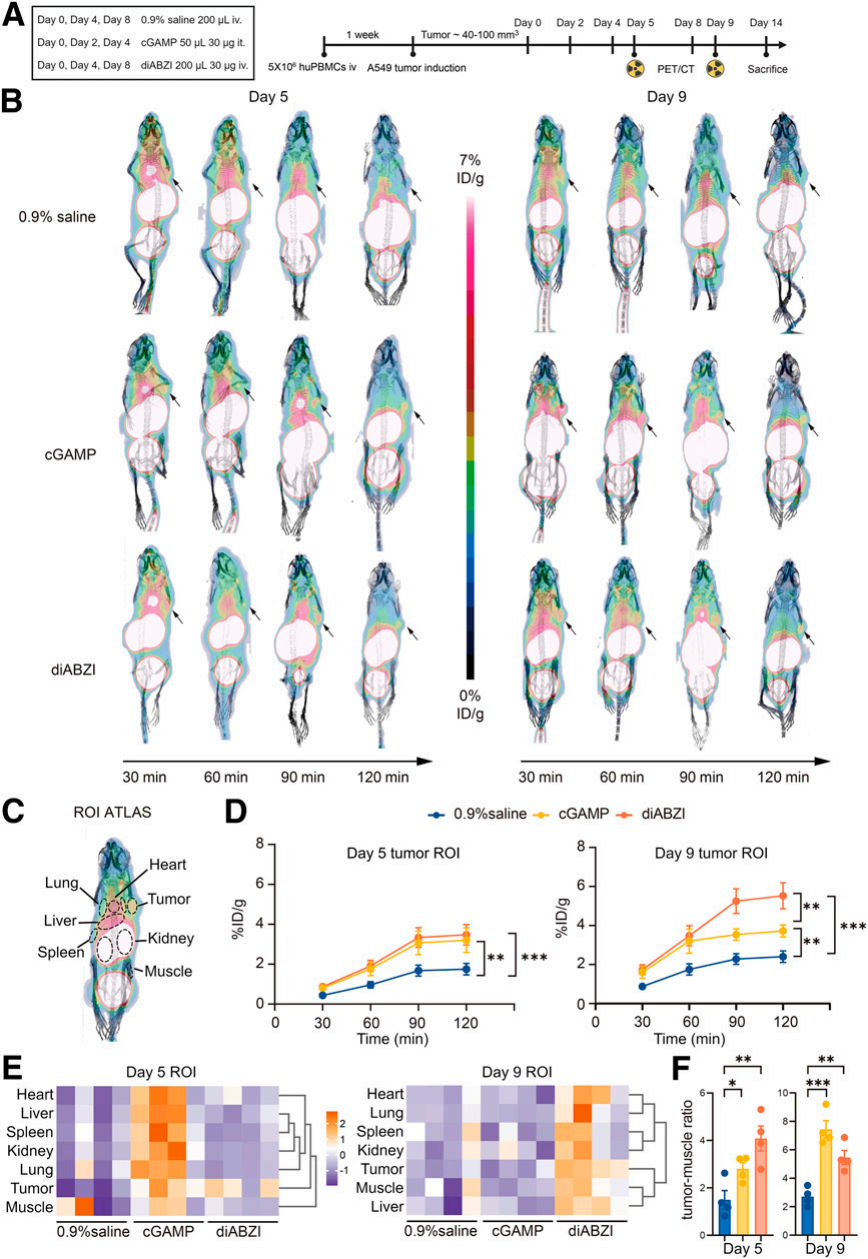
PET/CT imaging and ROI quantification of ^68^Ga-DOTA-ICOSpep in A549 CDX models. (A) Scheme of ICOS-targeting PET/CT imaging studies in humanized A549 tumor–bearing mice. (B) Representative small-animal PET/CT images of mice treated with 0.9% saline, cGAMP, or diABZI, acquired at multiple time points. Arrows indicate tumor locations. Image intensity is scaled to maximum of 7 %ID/g. (C) Schematic representation of 3-dimensional ROI used for quantitative image analysis. (D) Quantitative tracer uptake in tumors at 30, 60, 90, and 120 min postinjection. (E) *z* score normalized heatmap showing tracer uptake across selected ROIs. Rows are ordered using unsupervised hierarchical clustering on day 5 and day 9. ROI quantification in heart, liver, spleen, lungs, kidneys, tumor, and muscle on day 5 and day 9. (F) Quantitative tumor-to-muscle signal ratios after injection of ^68^Ga-DOTA-ICOSpep. All values represent mean ± SEM unless otherwise specified. One-way ANOVA and unpaired 2-tailed Student *t* test were used for comparation of ROI values. *P < 0.05, **P < 0.01, and ***P < 0.001. huPBMCs = humanized peripheral blood mononuclear cells; iv = intravenous.

**FIGURE 5.**
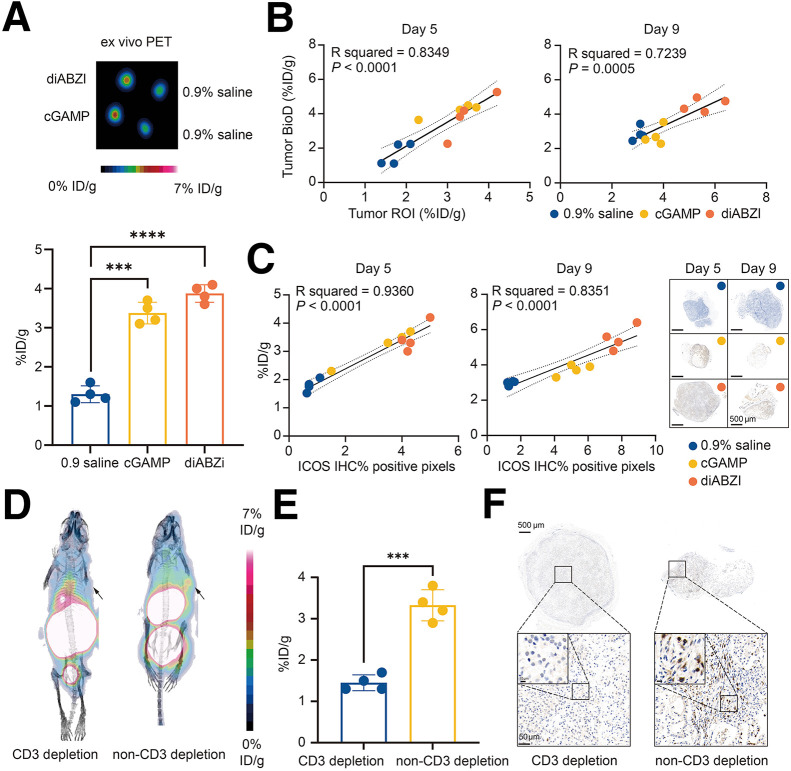
Validating specificity of ^68^Ga-DOTA-ICOSpep in detecting activated ICOS-positive T cells. (A) Representative ex vivo PET and PET/CT fusion images of excised tumors. (B) Linear regression analysis comparing ROI-derived PET quantification and ex vivo biodistribution data (BioD) at day 5 and day 9. (C) Correlation between percentage of positively stained pixels per field of view in immunohistochemistry (IHC) and tumor PET ROI uptake. (D and E) ICOS-targeted PET image showing elevated tracer uptake in tumors (arrows) in non–CD3-depleted versus CD3-depleted mice. (F) Immunohistochemistry staining of tumor sections revealed increased infiltration of ICOS-positive T cells in non–CD3-depleted mice. All values represent mean ± SEM unless otherwise specified. One-way ANOVA and unpaired 2-tailed Student *t* test were used for comparation of ROI values. ***P < 0.001 and ****P < 0.0001.

PET/CT imaging was performed on days 5 and 9 after tracer injection to assess the capability of ^68^Ga-DOTA-ICOSpep in capturing ICOS-positive activated T cells in vivo. PET images across all time points examined are presented in Figures 4A and 4B and Supplemental Figure 4A. Quantitative analysis was performed by drawing circular regions of interest (ROIs) over key metabolic organs, immune-related organs, and tumors (Fig. 4C). At day 5, more PET probes accumulated in the cGAMP (3.200 ± 0.3109 %ID/g) and diABZI groups (3.475 ± 0.2562 %ID/g), compared with that in the 0.9% saline group (1.750 ± 0.1443 %ID/g) (*P* < 0.01 and *P* < 0.001, respectively). At day 9, tumor ROI quantifications from cGAMP (3.725 ± 0.1548 %ID/g) and diABZI groups (5.525 ± 0.3351 %ID/g) were still higher than that of the 0.9% saline group (2.400 ± 0.1472 %ID/g) (*P* < 0.01 and *P* < 0.001, respectively) (Fig. 4D). ROI quantifications from all organs drawn are listed in Figure 4E. After *z* score normalization of ROI uptake values for each organ, unsupervised hierarchical clustering was performed to group tissues with similar tracer uptake distributions into the same cluster. According to the PET quantification profiles, the STING agonist–treated groups could be clearly distinguished from the phosphate-buffered saline cohort, indicating ^68^Ga-DOTA-ICOSpep PET imaging could be a robust strategy for identifying individuals benefiting from immunotherapy. Additional tumor-to-muscle ratio analysis was consistent with tumor ROI quantifications (Fig. 4F). PET imaging data from tumor regions was in line with fluorescence-activated cell sorting studies, indicating that ^68^Ga-DOTA-ICOSpep could precisely detect ICOS-positive activated T cells in vivo. To further confirm the accuracy of PET imaging studies, ex vivo tumor imaging and biodistribution studies (Supplemental Fig. 4B) were performed. Ex vivo ROI quantifications demonstrated clear and specific tracer uptake in the STING-treated groups, enabling excellent tissue discrimination (0.9% saline group: SUV_mean_, 2.87 ± 0.53; cGAMP group: SUV_mean_, 4.95 ± 0.47; diABZI group: SUV_mean_, 5.28 ± 0.13) (Fig. 5A). Linear regression analysis revealed that PET measurements of tumors perfectly fit biodistribution quantification (day 5: *R*^2^ = 0.8349, *P* < 0.0001; day 9: *R*^2^ = 0.7239, *P* = 0.0005) (Fig. 5B). Tumor ROI from both early and late stages had very strong linear correlation with ICOS-positive pixels in immunohistochemistry staining (day 5: *R*^2^ = 0.9360, *P* < 0.0001; day 9: *R*^2^ = 0.8351, *P* < 0.0001) (Fig. 5C). To further validate the specificity of ^68^Ga-DOTA-ICOSpep in detecting ICOS-positive T cells, CD3 T cells were depleted by intraperitoneal injection of 200 µg of antihuman CD3 antibody. The PET signal in tumor regions decreased in the CD3 depletion groups compared with the non-CD3 depletion group (*P* < 0.001) (Figs. 5D and 5E). Ex vivo immunohistochemistry results were consistent with PET imaging findings (Fig. 5F). Hematoxylin and eosin staining in major organs demonstrated high biocompatibility of the ICOS PET probes with both major metabolic and immune organs (Supplemental Fig. 4C).

### PET ROIs Correlate with Immunotherapy Response and Proinflammatory Profiles

**FIGURE 6.**
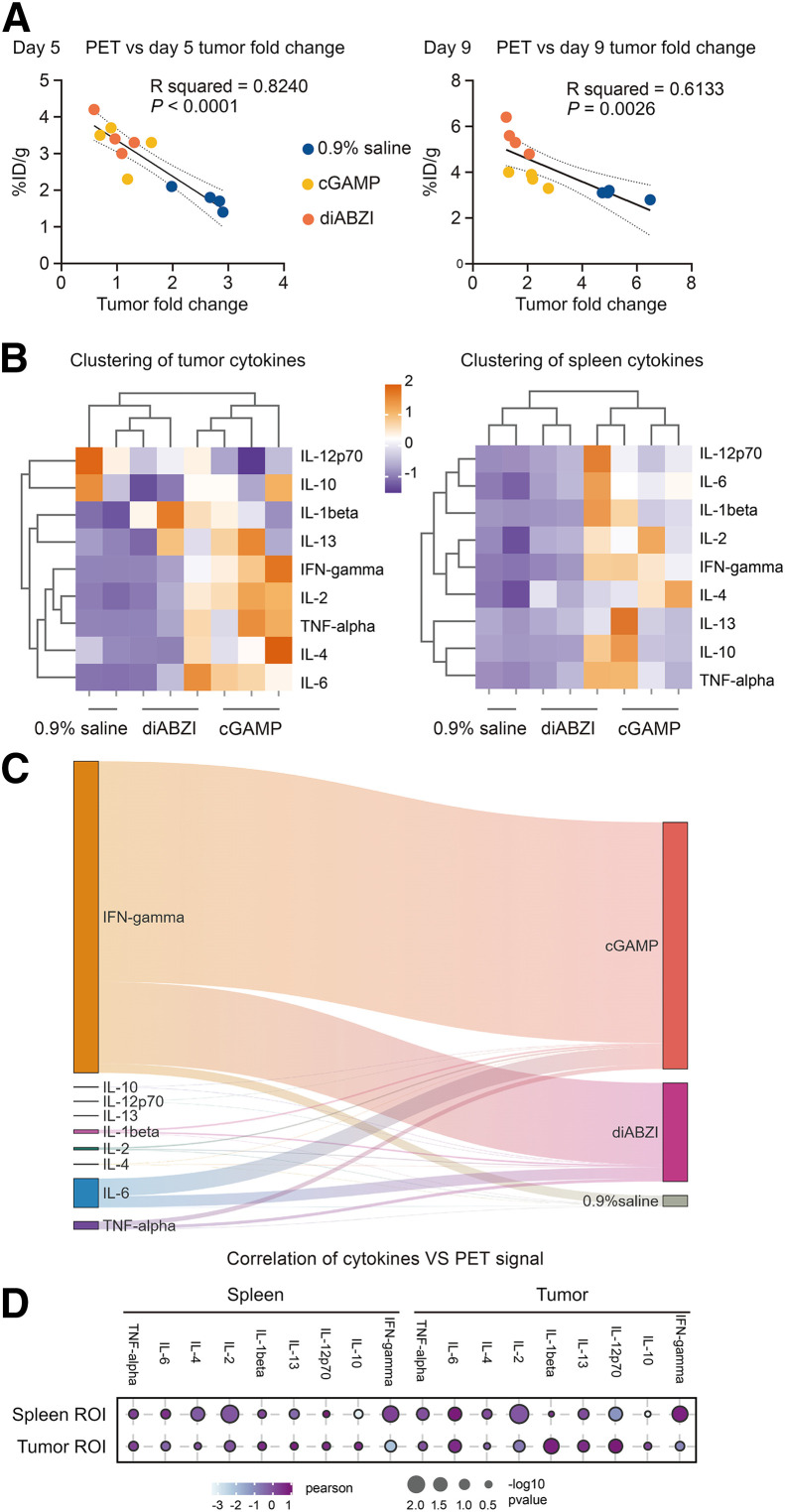
Prediction of immunotherapy response and proinflammatory profile by ICOS-targeted imaging. (A) Quantitative analysis of in vivo ICOS PET imaging correlated with tumor volume fold change. (B) Heatmap visualization of normalized cytokine concentrations across experimental groups. (C) Stacked bar chart showing the relative contribution of individual cytokines within each treatment group. (D) Bivariable heatmap illustrating Spearman correlation coefficients between cytokine levels and ICOS PET ROIs. All values represent the mean ± SEM unless otherwise specified. One-way ANOVA and unpaired 2-tailed Student *t* test were used for comparation of ROI values. IFN = interferon; IL = interleukin; TNF = tumor necrosis factor.

The major goal of this study was to evaluate whether ^68^Ga-DOTA-ICOSpep PET imaging could predict or monitor the therapeutic response of A549 mouse models to STING agonist treatment. Linear regression analysis demonstrated strong correlations between tumor volume fold change and PET signal (day 5 PET vs. day 5 tumor fold change: *R*^2^ = 0.8240, *P* < 0.0001; day 9 PET vs. day 9 tumor fold change: *R*^2^ = 0.6133, *P* = 0.0026), highlighting the potential of ICOS PET imaging for predicting and monitoring immunotherapeutic response (Fig. 6A). To better understand the immunologic features of the STING agonist, we tested the cytokines of spleen and tumor tissues via mesoscale discovery multiplex analysis. Unsupervised hierarchical clustering had distinguished STING agonist–treated groups from the 0.9% saline group, and elevated concentrations of proinflammatory cytokines were observed in the cGAMP- and diABZI-treated groups compared with the 0.9% saline control group (Fig. 6B; Supplemental Figs. 5A and 5B). The multifactor Sankey plot revealed that interferon-γ, interleukin-6, and tumor necrosis factor-α have a more dominating influence in STING pathways than do other cytokines (Fig. 6C). To further explore the utility of ICOS PET imaging in monitoring the proinflammatory environment, Spearman correlation coefficients between cytokine levels and ICOS PET ROIs were also evaluated. ICOS PET ROI quantifications from the spleen and tumors were highly correlated with certain proinflammatory cytokines examined (Fig. 6D). Overall, ICOS PET imaging could be an ideal approach for predicting and monitoring immune responses and the proinflammatory environment of cancer immunotherapy.

## DISCUSSION

Benefiting from the breakthroughs of immuno-oncology, immunotherapy has optimized clinical practice of cancer patient management. Given the complexity of the TME, therapeutic responses vary among patients. Thus, novel approaches need to be developed for precise assessment of immune responses. In this study, we identified ICOS as an indicator for assessing immune response. ^68^Ga-DOTA-ICOSpep PET imaging could precisely predict and monitor response induced via the STING agonist in a humanized lung cancer model.

We believe that the ICOS PET imaging probe could be used to overcome current challenges in lung cancer immunotherapy, and there are many compelling reasons for the ICOS PET imaging approach to be further translated into clinic.

T cell activation and proinflammatory cytokine release are key cellular events in cancer immunotherapy, with certain biomarkers upregulated on therapy initiation, and for PET imaging targets, there are many candidates for us to pick up with. Among all of these biomarkers, released cytokines might be diluted by blood flow, and on the other hand, T cell phenotype markers CD4 and CD8 could not distinguish activated and exhausted T cells (19); thus, we hypothesize T cell surface activation markers may be an ideal choice. Actually, different studies focusing on various T cell activation markers (ICOS, OX40, CD69, IL2RA, and CAD38) have already been reported (20–24). Previously, we compared the transcripts of these biomarkers on CD19 chimeric antigen receptor-T cells (13); most of them were upregulated early on T cell activation, but they all decreased at later stages. However, the unique marker ICOS maintained a high expression level across the whole T cell activation and cytotoxic killing process, making it a reliable imaging marker for longitudinal monitoring of immune responses in cancer immunotherapy. The omics data presented here from TCGA and Cancer Treatment Response gene signature databases demonstrated the variation of ICOS transcript levels between responders and nonresponders, as well as the excellent predictive performance of ICOS in lung cancer immunotherapy. Moreover, ICOS was also identified as a protective factor in patients with LUAD. As a conserved biomarker mainly expressed on activated T cells, ICOS upregulation was validated in multiple immunotherapy strategies and autoimmune diseases, including STING agonist, the programmed death protein-1/programmed death ligand-1 blockade, CD19 chimeric antigen receptor-T therapy, acute graft versus host disease, and rheumatoid arthritis (12,13,23,25). These findings emphasized the potential of ICOS as a robust imaging biomarker for monitoring immune responses, and relevant works have already been performed via a ^89^Zr-labeled monoclonal antibody targeting murine ICOS (12). Given the variation of ICOS structure between humans and rodents, this PET tracer failed in further clinical translation, which forced us to develop novel ICOS PET tracers for detecting human ICOS.

Our PET imaging studies in A549-CDX models demonstrated proof of concept how our ICOS imaging probe could be used to precisely capture ICOS-positive activated T cells and monitor the therapeutic response of cancer immunotherapy. ^68^Ga-DOTA-ICOSpep PET imaging could easily distinguish STING agonist–treated groups from a vehicle cohort at all time points examined, and this difference was eliminated by CD3 depletion, indicating the PET signal was T cell–dependent. The correlation between PET ROI quantifications and ICOS immunohistochemistry positive pixels, as well as biodistribution results, provided more relevant evidence about the accuracy and specificity of this PET tracer in detecting ICOS-positive activated T cells in vivo. Linear regression models between tumor PET ROI and volume fold change from various time points highlighted the capability of this imaging approach to predict and monitor immune responses. Moreover, ICOS imaging could make the nonobjective immune response visualized as intuitionistic photos, and this was more valuable than anatomic imaging techniques only focusing on tumor volume changes. ICOS imaging may also be used in novel immunotherapy adjuvant development. For example, STING agonists are being explored as novel strategies for cancer immunotherapy, with increasing insights from current clinical trials (NCT00738387, NCT03937141, NCT04220866, NCT04109092, NCT05321940). Those clinical trials often failed because of the intratumoral method of drug administration, which presents technical challenges and limits its application to patients with inaccessible solid tumors (26). Thus, the small-molecule STING agonist diABZI which is suitable for intravenous administration was developed for overcoming those challenges (27,28). In our PET study, the late time point PET ROI quantifications demonstrated the lower strength of immune responses in the cGAMP group, compared with that in the diABZI cohort, demonstrating the potential of this imaging approach for comparing the therapeutic effects from different drugs (29). Thus, long-time survival experiments could be optimized via a single noninvasive imaging study. Beyond in vivo visualization of ICOS-positive activated T cells, the strong correlation between PET ROI quantifications and cytokine levels highlighted the potential of ICOS PET imaging strategy in reflecting cytokine release and inflammatory responses within the TME and peripheral immune organs. Instead of 9–12-wk CT or MR scans, ICOS PET imaging could streamline the whole process of clinical patient management by monitoring the dynamics of biologic changes within tumors early after immunotherapy initiation, helping the physicians to make decisions on drug regimen selection or dose adjustment. Moreover, as the affinity of the ICOS-targeting peptide is currently in the submicromolar range, further optimization holds promise for developing more sensitive probes for ICOS imaging. Thus, the ICOS imaging strategy may improve patient outcomes by identifying true responders to cancer immunotherapy, as well as discarding the redundant therapies without any contribution to patient health. The kidneys would be a dose-limiting organ in human studies, with high renal retention raising possible dosimetry issues. To ensure safe clinical translation and to minimize radiation-induced nephrotoxicity, a detailed dosimetry study must be performed to precisely calculate the maximum permissible injection dose. In subsequent clinical development, we will establish the correlation between renal absorbed dose and injected activity using human biodistribution data and dosimetric models, ensuring that kidney exposure remains well below safety thresholds while maintaining sufficient image quality.

## CONCLUSION

Overall, in this proof-of-concept work, we identified ICOS as a sensitive and robust imaging biomarker for assessing therapeutic effects of lung cancer immunotherapy, and we also developed a novel peptide-based PET tracer, ^68^Ga-DOTA-ICOSpep, for detecting human ICOS-positive activated T cells. ^68^Ga-DOTA-ICOSpep PET imaging offers a convincing strategy for precise prediction and monitoring of immune responses. On the basis of the initial findings listed here, we believe that this imaging approach warrants further investigation in clinical trials.

## DISCLOSURE

Funding for this study was provided by the National Natural Science Foundation of China (grant nos. 82272056, 82472036, and 22174119), the Joint Fund of Zhejiang Provincial Natural Science Foundation of China (grant no. ZCLKLZ25H1602), the Ningbo Major Research and Development Plan Project (grant no. 2024Z214), the Ningbo Natural Science Foundation (grant no. 2024J043), the Ningbo Yongjiang Talent Program (2023), The Heilongjiang Postdoctoral Scientific Research Developmental Fund (grant no. LBH-Q21136), and the Natural Science Foundation of Heilongjiang Province (grant no. LH2023H045). No other potential conflict of interest relevant to this article was reported.

## Notes

KEY POINTS **QUESTION:** Despite the imaging technologies currently applied in the clinic, is there any promising strategy capable of precisely predicting immune responses among patients with lung cancer? **PERTINENT FINDINGS:** The ICOS peptide PET tracer, ^68^Ga-DOTA-ICOSpep, was capable of properly capturing ICOS-positive activated human T cells in vivo. ICOS PET imaging successfully predicted the immune response and cytokine release induced by STING agonists. **IMPLICATIONS FOR PATIENT CARE:** Future clinical translation of imaging ICOS peptides is predicted to enable noninvasive and exact querying of T cell activity in patients, potentially improving the capacity of lung cancer immunotherapy to predict treatment response.
